# Assessment of Knowledge and Attitude towards Palliative Care and Associated Factors among Nurses Working in North Wollo Hospitals

**DOI:** 10.4314/ejhs.v31i2.22

**Published:** 2021-03

**Authors:** Addisu Getie, Adam Wondmieneh, Ayelign Mengesha, Awet Fitwi, Getnet Gedefaw, Asmamaw Demis

**Affiliations:** 1 Department of nursing, college of health science, Woldia University, Woldia, Ethiopia; 2 Department of midwifery, college of health science, Woldia University, Woldia, Ethiopia

**Keywords:** Knowledge, attitude, palliative care, nurses

## Abstract

**Background:**

Palliative care improves the quality of life of patients facing a life-threatening illness. Nurses should improve their caregiving capacity. In Ethiopia, palliative care is underestimated. The availability of data regarding the knowledge and attitude of nurses towards palliative care is critically important. Thus, this study aimed to assess the level of knowledge and attitude of nurses towards palliative care.

**Methods:**

Institution-based, cross-sectional study was conducted in North Wollo hospitals. A simple random sampling technique was used. The data was collected using structured self-administered questionnaires from February to March 2019. The analysis was done using a binary logistic regression model. P-value < 0.05 was considered as statistically significant.

**Results:**

The result revealed that 59.7% of the respondents had good knowledge and 44.2% had a favorable attitude towards palliative care. Level of education, experience in caring for chronically ill patients, and experience in caring for dying family members within the last 6 months had a significant association with the knowledge of nurses. Monthly income, experience in caring for chronically ill patients, formal palliative care education, and knowledge were found statistically significant with the attitude of nurses towards palliative care.

**Conclusion:**

More than half of the nurses had good knowledge, but less than half of the respondents had a favorable attitude towards palliative care. Attention should be given towards palliative care by the health policy and needs to be incorporated into the national curriculum of nursing education.

## Introduction

Palliative care is organized care that is provided to patients and their families who have progressive, chronic, and life-threatening disease to relieve the symptoms by giving physical, psychosocial, and spiritual care (1). It improves the quality of life of patients facing life-threatening illness through the prevention and relief of suffering utilizing focused assessment, early problem identification, and relieving of pain (2). Worldwide, over 29 million people died from diseases requiring palliative care. The estimated number of people in need of palliative care at the end of their life was 20.4 million. Of all people who need palliative care, 94% are adults; among these, 69% were greater than 60 years old, and 25% were between 15 to 59 years old. Of these people in need of palliative care, 78% live in low- and middle-income countries (4).

Globally, 40–60% of all deaths need palliative care. The majority of adults who need palliative care have chronic diseases such as cardiovascular diseases, cancer, chronic respiratory diseases, AIDS, and diabetes mellitus. Pain is one of the most frequent and serious symptoms experienced by patients in need of palliative care. Since the early 1980s, the need for palliative care has been progressively acknowledged worldwide. However, many patients still require palliative care in most parts of the world (4). Palliative care is a new concept in developing countries and lacks in most African countries including Ethiopia. Applying palliative care faced great challenges in the developing world due to a lack of trained health professionals like nurses and poor understanding of palliative care among healthcare providers (5).

Ethiopia is attempting to develop a policy for establishing palliative care. However, most people are suffering from chronic illnesses that need palliative care (6). Since patients who need palliative care are increasing as a result of raised chronic diseases, nurses should prepare themselves to give palliative care (7,8). Nurses with a low level of knowledge about palliative care do not skillfully assess the patients' needs and are not competent to develop effective communication with chronically sick patients and their families (8). A negative attitude towards palliative care is also a barrier to appropriate palliative care. This unfavorable attitude prevents nurses' from involving in accepting and giving care to patients suffering from chronic disease (9).

Understanding risk factors and prognostic factors for knowledge and attitude of nurses in low/middle-income countries is an important issue that may differ from high-income settings. As a result, knowing the level of knowledge and attitude of nurses towards palliative care may give an insight into where interventions and resources should be focused as well as having an understanding of the burden of disease in low-income countries like Ethiopia. Several patients need palliative care. However, the issue of nurses' knowledge and attitude is poorly addressed. Therefore, this study aimed to identify the level of knowledge and attitude of nurses towards palliative care in North Wollo hospitals.

## Methods

**Study setting and design**: North Wollo is located in Amhara region with the capital city of Woldia found 521 KM away from Addis Ababa and 360km from Bahirdar. There are five governmental health hospitals in North of Wollo. These are Woldia Hospital, Kobo Hospital, Lalibela Hospital, Mekiet Hospital, and Wadila Hospital. Five hundred seventy-two nurses are working in these five hospitals. Of these, 266 were BSc nurses and 306 were diploma nurses. An institution-based cross-sectional study was conducted from April 1 - 15, 2019, in north Wollo governmental hospitals.

**Study population**: All nurses working in North Wollo governmental hospitals were recruited for this study. Nurses with work experience of at least 6 months and who were working during the study period were included in the study whereas nurses who were on annual leave, maternal leave, and unavailable due to short-term and long-term training were excluded from the study.

**Sample size determination**: The sample size was determined using a single population proportion formula designated as (n= (Zα/2)2p (1-p)/d2)); where n: the required minimum and feasible sample size, Za/2(1.96): significance level at α=0.05 with 95% confidence interval, p: proportion of the level of knowledge on palliative care used in Addis Ababa public hospitals (30.5%) (14), and d: margin of error (5%). Based on the assumptions of the single proportion formula, the calculated sample size became 226. In addition, the assumption of correction formula was carried out due to the study population was less than 10,000. After all, by considering 10% of the non-response rate, the final sample became 229.

**Sampling technique and sampling procedure**: All hospitals found in the North Wollo Zone were included in the study. Amongst the total number of nurses (572) working in North Wollo hospitals, 229 nurses were recruited from five hospitals through the proportional sample allocation technique.

**Figure 1:**
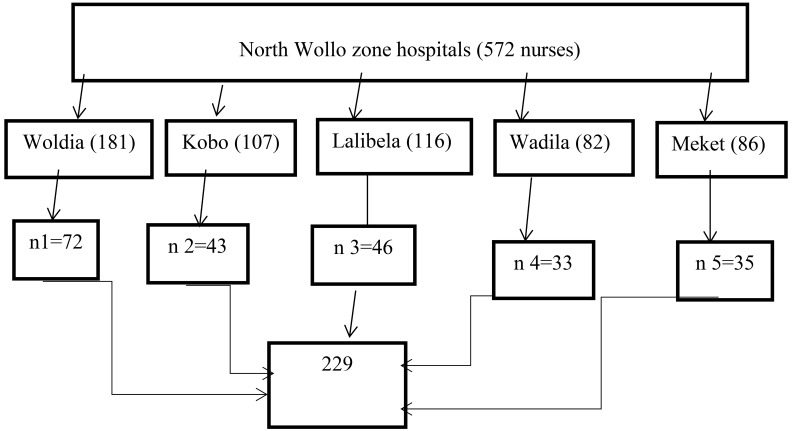
Sampling procedure of nurses working in north Wollo governmental hospitals, 2019

Each study participant (Nurses) was selected via a computer-generated based simple random sampling technique (Figure 1).

**Data collection**: Data were collected using self-administered questionnaires consisted both open and closed-ended questions. The questioner consists of information on socio-demographic characteristics, knowledge and knowledge related factors, attitude & attitude related factors of nurses towards palliative care. Five BSc nurses and three MSc nurses were recruited for data collection and supervision for the study respectively. The questioners were adapted and modified from different articles (10, 11, 14). Knowledge and attitude of nurses were assessed using a mean score, through validated questioner using pre-test. There were twenty (20) items used to assess the level of knowledge of nurses with a reliability coefficient of 0.87 and nineteen (19) items to assess the attitude of nurses with a reliability coefficient of 0.86.

**Data quality control**: Different methods of data quality control were used. A structured and validated questionnaire was used. Pretest was carried out among 11(8.5%) of the sample size in Tefera Hailu Memorial Hospital found in Waghimra Zone. Based on the findings of the pretest, some modifications and adjustments were done for the data collection tool. One day training was provided for the data collectors and supervisors regarding the data collection procedure and tool. Close supervision was conducted by the supervisors regarding the data collection process. The clarity and completeness of each item of the questionnaire were checked.

**Ethical approval and consent to participate**: Ethical clearance was obtained from the institutional review board of Woldia University. An official letter from the College of Health Science was written to the Woldia City Administration health bureau and the selected hospitals. Permission was obtained from Woldia City Administration Health Bureau and an official letter was distributed to the selected hospitals. Informed written consent was also obtained from all study participants after the information is provided about the purpose of the study, non-invasiveness of the data collection procedure. Confidentiality of the personnel data was reassured. The study participants had the right to ask anything about the study and the right to refuse participation in the study at any moment they want.

**Data processing and analysis**: After the data collection, a data template format was prepared, coded, and entered into Epi Data version 4.2. Then, the data was exported to SPSS version 24 for analysis. Descriptive analysis was employed to describe the percentages and distributions of the respondents' socio-demographic characteristics and the factors that influence clinical palliative care of Nurses and presented in the form of tables, graphs, and charts. Bivariable and multivariable analyses were used to assess the association between the explanatory variable with the outcome variable. A crude and adjusted odds ratio with the corresponding 95% confidence interval was also computed. P-value <0.05 was declared as statistically significant.

## Results

Of 229, 226 participants responded completely which gives a 98.7% response rate. Non-response was due to unwillingness. Among the 226 nurses who completed the questionnaire, the majority,132 (58.4%), were males and 94 (41.6%) were females. The mean age of the respondents was 28.55 years ± 5.752 SD (Table 1).

**Table 1:** Socio-demographic character of nurses in North Wollo Zone hospitals, 2019

Variables	Response	Frequency	Percentage
Age classification	21–30	166	73.5
	31–40	45	19.9
	>40	15	6.6
Monthly income	<=3000 Birr	36	15.9
	>3000 Birr	190	84.1
Marital status	Single	87	38.5
	Married	133	58.8
	Divorced	6	2.7
Level of education	Diploma	93	41.2
	BSc nurse	133	58.8
Current working unit	Medical	48	21.2
	Surgical	23	10.2
	Pediatrics	27	12
	Obstetrics/gynecology	33	14.6
	OPD	50	22.1
	Emergency	45	20
Work experience	<6 years	145	64.2
	6–10 years	65	28.8
	11–15 years	9	4
	>15 years	7	3.1

Of all respondents, 19.5% of study subjects did not have any experience in caring for chronically ill patients while only 26.1% had in-service training about palliative care (Table 2).

**Table 2:** Clinical characteristics of nurses work in North Wollo Zone hospitals, 2019

Variables	Response	Frequency(N)	Percentage (%)
Experience caring chronically patient	Daily	80	35.4
	Once / week	27	11.9
	Once /month	39	17.3
	Few times per year	36	15.9
	Never	44	19.5
Experience caring for dying family	Yes	85	37.6
member in the last 6 month	No	141	62.4
Learned formal palliative care in	Yes	141	62.4
college/university	No	85	37.6
Take in-service training about palliative	Yes	59	26.1
care	No	167	73.9

**Nurses' Knowledge and attitude towards palliative care**: The result of this study showed that 59.7% of nurses had good knowledge, whereas 40.3% had poorly knowledgeable. Regarding attitude, 44.2% of respondents had a favorable attitude, whereas 55.8% of respondents had an unfavorable attitude.

**Knowledge-related factors towards palliative care**: As illustrated in Table 3, the nurses' knowledge towards palliative care was affected by the level of education, experience in caring for a chronically ill patient, and the experience of caring for the dying family member within the last 6 months. Nurses who were BSc holders were 2.34 times more likely to be knowledgeable than nurses who were diploma holders [AOR: 2.34; 95% CI (1.19–4.61)]. Nurses who had daily experience in caring for a chronically ill patient were 4.74 times more likely to be knowledgeable than those who did not have experience in caring for a chronically ill patient [AOR: 4.74; 95% CI (1.82–12.38)].

**Table 3:** Factors affecting nurses' knowledge of palliative care in North Wollo Zone hospitals, 2019

Variables		Knowledge		COR(95%CI)	AOR(95%CI)	P-value
					
		Good N (%)	Poor N (%)			
Institution /hospital	Primary	97(62.6)	58(37.4)	1.45(0.82–2.57)	1.29(0.65–2.55)	0.46
	General	38(53.5)	33(46.5)	1	1	
Age	21–30	105(63.3)	61(36.7)	1.97(0.68–5.69)	0.61(0.26–1.4)	0.24
	31–40	23(51.1)	22(48.9)	1.2(0.37–3.852)	1.4(0.35–5.56)	0.63
	>40	7(46.67)	8(53.33)	1	1	
Education	BSc	88(66.12)	45(33.88)	1.91(1.11–3.29)	2.34(1.19–4.61)	0.014
	Diploma	47(50,54)	46(49.46)	1	1	
Experience caring	Daily	55(50.00)	25(50.00)	3.85 (1.78–8.36)	4.74 (1.82–12.36)	0.001
for chronically ill	Once /week	19(70.37)	8(29.63)	4.16 (1.49–11.64)	3.21 (1.48–18.31)	0.010
patients	Once /month	24(61.54)	15(38.46)	2.8(1.15–6.82)	2.79 (0.95–8.21)	0.063
	Few time/year	21(58.33)	15(41.67)	2.45(0.993–6.05)	1.92 (0.63–5.84)	0.250
	Never	16(36.36)	28(63.64)	1	1	
Experience caring	Yes	68(80)	17(20)	4.42 (2.36–8.26)	5.04 (2.33–10.89)	0.0001
dying patient	No	67(47.52)	74(52.48)	1	1	
Formal palliative	Yes	96(68.09)	45(31.91)	2.52 (1.45–4.38)	1.36 (0.66–2.79)	0.409
care education	No	39(45.88)	46(54.12)	1	1	

* AOR: Adjusted Odds Ratio, CI: Confidence Interval, COR: Crude Odds Ratio

Nurses who had experience in caring for a chronically ill patient at least once per week were 3.21 times more likely to be knowledgeable than those who did not have experience in caring for a chronically ill patient [AOR: 3.21; 95% CI (1.48–18.31)]. Nurses who had an experience of caring for the dying family members within the last 6 months were 5.04 times more likely knowledgeable than those who had not to experience [AOR: 5.04; 95% CI (2.33–10.89)] (Table 3)

**Attitude-related factors towards palliative care**: As illustrated in Table 4, the nurses' attitude towards palliative care was affected by monthly income, work unit, experience in caring for chronically ill patients, having formal palliative care, education, and knowledge. Nurses whose monthly income was greater than 3000 Ethiopian Birr were 3.21 times more likely to have a favorable attitude towards palliative care than whose monthly income was less than 3000 birr [AOR: 3.21; 95% CI (1.33–7.74)].

**Table 4:** Factors affecting nurses' attitude towards palliative care in North Wollo Zone hospitals, 2019

Variables		Attitude		COR(95%CI)	AOR(95%CI)	P-value
					
		Positive N (%)	Negative N (%)			
Income	>3000	88(46.32)	102(53.68)	1.73(1.816–3.65)	3.21(1.33–7.74)	0.009
	<=3000	12(33.33)	24(66.66)	1	1	
Experience caring for chronically ill patients	Daily	43(53.75)	37(46.25)	4.52(1.92–10.62)	4(1.5–10.67)	0.006
	Once /week	13(48.15)	14(51.85)	3.61(1.26–10.34)	3.94(1.16–13.43)	0.028
	Once /month	19(48.72)	20(51.28)	3.69(1.41–9.97)	3.48(1.14–10.67)	0.029
	Few time/year	16(44.44)	20(55.56)	3.11(1.16–8.33)	2.97(0.94–9.15)	0.064
	Never	9(20.45)	35(79.55)	1	1	
Formal palliative care education	Yes	71(71)	29(29)	1.96(1.12–3.42)	2.37(1.22–4.63)	0.011
	No	70(55.56)	56(44.4)	1	1	
Knowledge	Good	72(53.33)	63(46.67)	2.57(1.47–4.497)	2.89 (1.49–5.6)	0.002
	Poor	28(30.77)	63(69.23)	1	1	

* AOR: Adjusted Odds Ratio, CI: Confidence Interval, COR: Crude Odds Ratio

Nurses who had daily experience in caring for a chronically ill patient were 3.99 times more likely to have a favorable attitude than those who did not have experience in caring for a chronically ill patient [AOR: 3.99; 95% CI (1.5–10.67)]. Nurses who had experience in caring for a chronically ill patient at least once per week were 3.94 times more likely to have a favorable attitude than those who did not have experience in caring for a chronically ill patient [AOR: 3.94; 95% CI (1.56–13.43)]. Nurses who had experience in caring for a chronically ill patient at least once per month were 3.48 times more likely to have a favorable attitude than those who did not have experience in caring for a chronically ill patient [AOR: 3.48; 95% CI (1.14–10.66)]. Nurses who learned formal palliative care were 2.37 times more likely to have a favorable attitude than those who had not [AOR: 2.37; 95% CI (1.22–4.63). Nurses who had good knowledge were 2.89 times more likely to have a favorable attitude than those who had poor knowledge [AOR: 2.89; 95% CI (1.49–5.6) (Table 4).

## Discussion

This study assesses the knowledge and attitude of nurses towards palliative care. The knowledge of nurses towards palliative care was not enough to give appropriate nursing care for patients who require palliative care. This knowledge deficiency might be due to serious deficiencies in the undergraduate nursing education curriculum which does not incorporate palliative care as it is needed (7,11). Even if this finding regarding nurses' knowledge is not enough to give care, it is higher than the findings of studies done in Palestine (20%), India (21%), Saudi Arabia (38%), and Addis Ababa (30.5%, 26.5%) (7,8,12,13,14). This might be due to the difference in the study period that the previous studies were conducted before five years when in-service training and formal palliative care education were not adequate. However, it is lower than a study conducted in Mangalore, India (72%) (15). It might be due to the difference in the study setting in which the previous study was done in a high-income country where nurses can get different access to update their knowledge about palliative care. From this study, the level of education, experience in caring for a chronically ill patient, and experience in caring for dying family members within the last 6 months had a significant effect on the knowledge of nurses. This might be since education is a cornerstone for knowledge as BSc nurses had further education, in-depth training, and practices in health institutions which leads them to be more knowledgeable than diploma nurses. Regarding experience, being experienced in caring for patients who need palliative care is a good source of knowledge due to continual exposure with a patient. These findings are in line with a study conducted in Northern Districts, Palestine, which revealed that nurses' level of education and experience had a significant effect on the knowledge of nurses about palliative care (12). However, it disagrees with a study done in public hospitals in Addis Ababa. In the previous study, there is no association between the level of education and knowledge of nurses on palliative care (13,14). The possible reason for this discrepancy may be due to the difference in case flow in which the previous study was done in a more urbanized setting than the current study. Thus, in a highly urbanized setting, patient flow is higher than in a less urbanized setting. This shows that nurses working in hospitals having high patient flow may have experience in caring for chronically ill patients. There is a substantial deficiency regarding nurses' attitude towards palliative care in this study. This deficiency might be due to the reason of lack of in-service training, poor knowledge about palliative care, absence of formal palliative care education, and poor job satisfaction of nurses. The attitude of nurses towards palliative care in this study is lower than studies conducted in Saudi Arabia (83%), Palestine (62.5%), and Sere Gokulam Medical College (82.5%) (8,12,16). The possible reason for this difference may be due to the presence of curriculum education content about palliative care in previous study areas or the absence (inadequacy) of palliative care education in Ethiopia. Moreover, this study is almost similar to a study conducted in Addis Ababa in which 46.3% of nurses had a favorable attitude towards palliative care (9).

The findings of this study confirmed the association of monthly income, experience in caring for chronically ill patients, taking formal palliative care education, and knowledge with the attitude of nurses towards palliative care. Regarding monthly income, as monthly income increases, there will be job satisfaction, which indirectly leads to having a favorable attitude. This disagrees with studies done in Palestine, Addis Ababa, and Amhara region referral hospitals in which monthly income had no significant association with nurses' attitude towards palliative care (5,12,14). The discrepancy might be due to the difference in the study setting level in which the previous studies were done in referral hospitals but the current one was done in general and primary hospitals. Thus, nurses who work in referral hospitals may have additional income like overtime income other than a monthly salary. Nurses who had experience in caring for chronically ill patients had frequent contact and were knowledgeable, which leads them to have a more favorable attitude towards palliative care than those who had no experience at all. Nurses who took formal palliative care education were more knowledgeable than those who did not take it. This leads them to have a positive attitude. This is in line with the study done in Addis Ababa public hospitals and Amhara region referral hospitals regarding caring for chronically ill patients (5,14).

The result of this study suggested that more than half of the nurses had good knowledge but the majority of respondents had an unfavorable attitude towards Palliative care. The level of education and experience in caring for a chronically ill patient had a significant association with the knowledge of nurses. Monthly income, experience in caring for chronically ill patients, formal palliative care education, and knowledge were found to be statistically significant with the attitude of nurses towards palliative care. As a result, nurses should build their knowledge and attitude towards palliative care through basic in-service training and formal palliative care education.

As the study focuses on nurses, the finding would be used by the nurses to give attention to their patients who need palliative care. It can be used also as baseline information to research the practice of health professionals regarding palliative care. However, it was impossible to establish a cause and effect relationship as the study design was cross-sectional. Due to the small sample size, the result might not be representative of all nurses working in Ethiopia.
